# TmDOTA-Tetraglycinate Encapsulated Liposomes as pH-Sensitive LipoCEST Agents

**DOI:** 10.1371/journal.pone.0027370

**Published:** 2011-11-28

**Authors:** Ana Christina L. Opina, Ketan B. Ghaghada, Piyu Zhao, Garry Kiefer, Ananth Annapragada, A. Dean Sherry

**Affiliations:** 1 Department of Chemistry, University of Texas at Dallas, Richardson, Texas, United States of America; 2 School of Biomedical Informatics, University of Texas Health Sciences Center at Houston, Houston, Texas, United States of America; 3 Advanced Imaging Research Center, University of Texas Southwestern Medical Center, Dallas, Texas, United States of America; National Institutes of Health, United States of America

## Abstract

Lanthanide DOTA-tetraglycinate (LnDOTA-(gly)_4_^−^) complexes contain four magnetically equivalent amide protons that exchange with protons of bulk water. The rate of this base catalyzed exchange process has been measured using chemical exchange saturation transfer (CEST) NMR techniques as a function of solution pH for various paramagnetic LnDOTA-(gly)_4_^−^ complexes to evaluate the effects of lanthanide ion size on this process. Complexes with Tb(III), Dy(III), Tm(III) and Yb(III) were chosen because these ions induce large hyperfine shifts in all ligand protons, including the exchanging amide protons. The magnitude of the amide proton CEST exchange signal differed for the four paramagnetic complexes in order, Yb>Tm>Tb>Dy. Although the Dy(III) complex showed the largest hyperfine shift as expected, the combination of favorable chemical shift and amide proton CEST linewidth in the Tm(III) complex was deemed most favorable for future *in vivo* applications where tissue magnetization effects can interfere. TmDOTA-(gly)_4_^−^ at various concentrations was encapsulated in the core interior of liposomes to yield lipoCEST particles for molecular imaging. The resulting nanoparticles showed less than 1% leakage of the agent from the interior over a range of temperatures and pH. The pH *versus* amide proton CEST curves differed for the free *versus* encapsulated agents over the acidic pH regions, consistent with a lower proton permeability across the liposomal bilayer for the encapsulated agent. Nevertheless, the resulting lipoCEST nanoparticles amplify the CEST sensitivity by a factor of ∼10^4^ compared to the free, un-encapsulated agent. Such pH sensitive nano-probes could prove useful for pH mapping of liposomes targeted to tumors.

## Introduction

agnetic resonance imaging (MRI) is a powerful diagnostic tool for clinical imaging. The intensity of a proton MR signal is determined by numerous factors including tissue proton density, relaxation rates (T_1_ and T_2_), pulse sequence, blood flow and molecular diffusion effects. Paramagnetic MRI contrast agents are often used to enhance inherent contrast differences between tissues. Current clinical agents are largely based on paramagnetic gadolinium complexes which operate by shortening the relaxation times of the bulk water protons in their immediate vicinity [1]. A new class of agents called chemical exchange saturation transfer (CEST) agents has recently emerged that alter image contrast by reducing the total water signal intensity via chemical exchange of pre-saturated proton spins into the pool of water. The first CEST agents introduced by Balaban and co-workers [2], [3], [4] were diamagnetic molecules containing NH and/or OH protons that exchange with bulk water protons. Later, paramagnetic CEST agents (PARACEST) were shown to have some advantages over diamagnetic agents because the exchange sites in these agents tend to be shifted well away from the tissue water frequency so they are more readily saturated without inadvertent saturation of tissue water itself [5], [6]. A large chemical shift difference is also advantageous in that faster exchanging systems can be used for CEST imaging. Although Bloch theory predicts that a PARACEST agent with the optimal exchange rate will have comparable sensitivity to conventional Gd(III)-based T_1_ contrast agent, the number of such agents identified to date with exchange rates optimal for CEST are limited.

One way of magnifying CEST sensitivity is through the use of nano-systems which can sequester high concentration of exchangeable CEST sites within a small volume. Liposomes, a class of nano-systems, are spherical vesicles made up of one or more phospholipid bilayers. The phospholipid bilayer serves to separate the aqueous internal core of the liposomes from the surrounding medium. Liposomes provide a good platform for CEST since the encapsulated internal water protons can exchange with the external water protons by diffusion through the semi-permeable bilayer. Aime and co-workers have taken advantage of this signal enhancement effect by encapsulating a water shift reagent, Tm-DOTMA (not a PARACEST agent), in liposomes to shift the resonance frequency of the encapsulated water molecules away from the frequency of bulk water [7]. The entrapped, shifted water proton resonance then serves as a strong saturation antenna to initiate CEST. The resulting lipoCEST particles extend the sensitivity of CEST into the sub-nanomolar range.

It has been demonstrated that LnDOTA-(gly)_4_^−^ contains two chemical types of exchange sites, the Ln(III)-bound water protons and the amide protons [8]. Terreno et al., examined the CEST properties of these complexes previously and paid particular attention to the pH sensitivity of the amide protons in this series [9]. They proposed that one could use a combination of two different LnDOTA-(gly)_4_^−^ complexes, one showing favorable CEST from a slowly exchanging bound water molecule (insensitive to pH) and another showing favorable CEST from the highly shifted –NH protons (sensitive to pH), to establish a ratiometric method for imaging the pH of solution. Although promising, the relatively low sensitivity of CEST from any small molecule agent remains the major limitation for *in vivo* studies. In this work, we explored the potential of encapsulating LnDOTA-(gly)_4_^−^ inside liposomes as a method to enhance CEST sensitivity. LnDOTA-(gly)_4_^−^ complexes are highly water soluble, are relatively non-toxic in animals [10], and can be loaded into liposomes without the need for further chemical modification.

## Results

**Figure 1:**
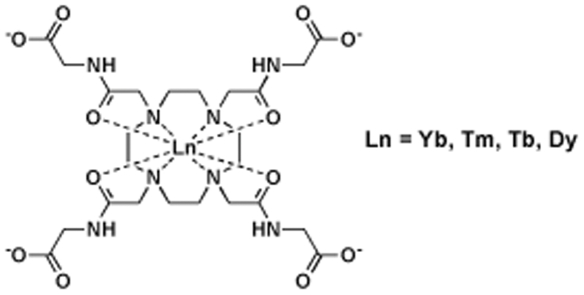
Molecular structure of LnDOTA-tetraglycinate (LnDOTA-(gly)_4_^−^ where Ln refers to the indicated lanthanide(III) ions).

**Figure 2:**
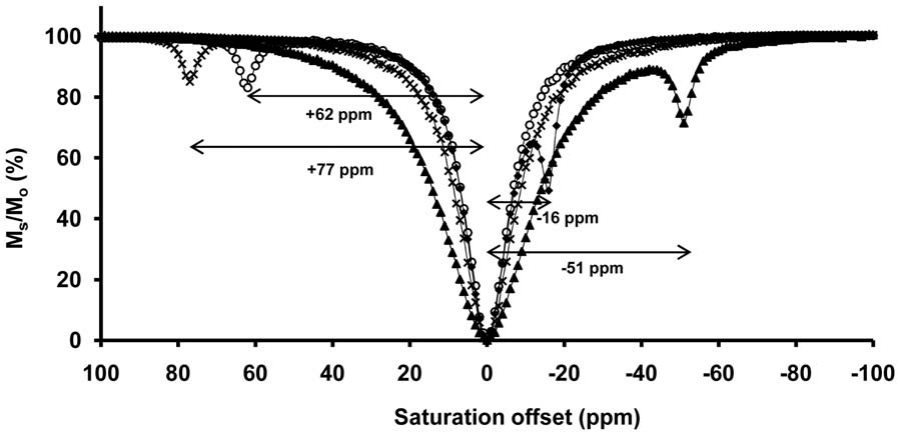
CEST spectra of the four LnDOTA-(gly)_4_ complexes. Each complex (20 mM, pH 7.5, 310 K) shows a different amide proton chemical shift with respect to the bulk water (δH_2_O = 0 ppm). The spectra were collected at 9.4 T using a frequency-selective, presaturation pulse over the frequency range ±100 ppm (B_1_ = 21.2 µT, 2 s for Tm-, Dy-, Tb-DOTA-(gly)_4_^−^ and 4 s for YbDOTA-(gly)_4_^−^).

The lanthanide complexes of DOTA-(gly)_4_ (Fig. 1) form a single species in solution whose conformation corresponds to a mono-capped square antiprism (SAP). The ^1^H-NMR of the LnDOTA-(gly)_4_^−^ matched perfectly with a spectrum published previously [8]. The CEST spectra of the four lanthanide complex recorded are compared in Fig. 2. The spectra were obtained by stepping the frequency of the presaturation pulse from +100 ppm to −100 ppm while monitoring the effects of saturation on the water amplitude. The magnitude of the water proton intensity normalized to 1 (M_s_/M_o_) is then plotted against the saturation offset at constant power. Pre-saturation of the bulk water at 0 ppm reduced M_s_/M_o_ to ∼0% in all samples. However, a second CEST exchange peak was observed in each spectrum that could be assigned to –NH proton exchange. These appeared at +77 ppm, +62 ppm, −16 ppm and −51 ppm for Dy-, Tb-, Yb- and TmDOTA-(gly)_4_^−^, respectively.

**Figure 3:**
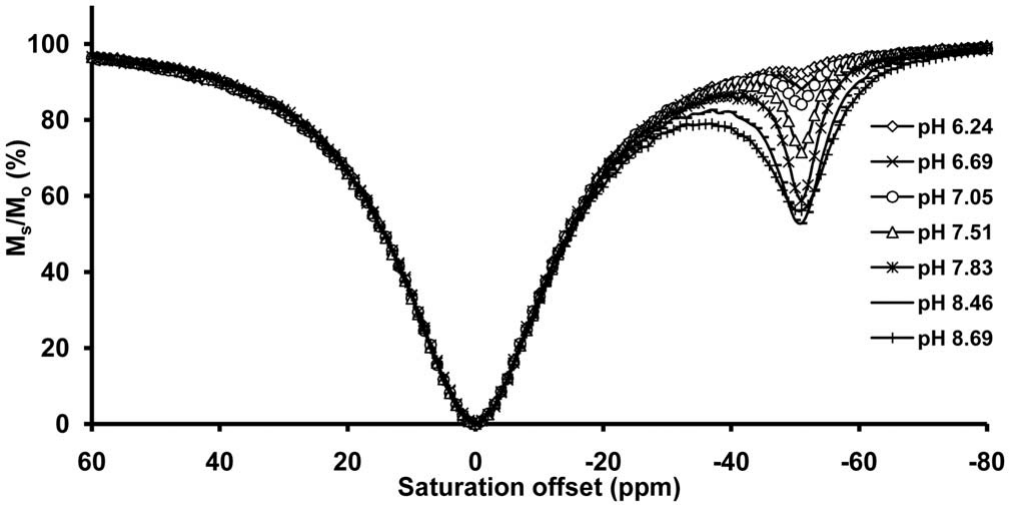
CEST spectra of TmDOTA-(gly)_4_^−^ collected at different pH values. Each spectrum was recorded at 9.4 T on 20 mM samples at pH 7.5 and 310 K. (B_1_ = 21.2 µT, presaturation time = 2 s).

**Figure 4:**
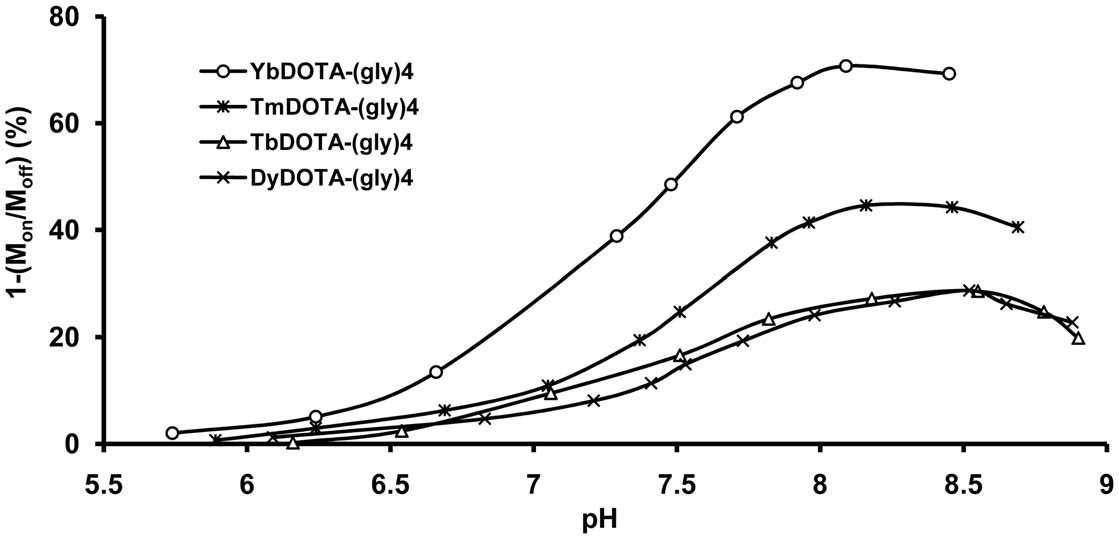
Amide proton CEST intensity *versus* pH for the four different LnDOTA-(gly)_4_^−^ complexes. Each amide proton CEST intensity was recorded on 20 mM samples at 9.4 T, 310 K and the indicated pH after application of a frequency-selection presaturation pulse set to the frequency of the exchanging –NH proton characteristic of each complex (B_1_ = 21.2 µT, presaturation time = 4 s (YbDOTA-(gly)_4_^−^) or 2 s (TmDOTA-(gly)_4_^−^, TbDOTA-(gly)_4_^−^, DyDOTA-(gly)_4_^−^).

The CEST dependence on pH was also evaluated for each of the four LnDOTA-(gly)_4_^−^ complexes. The CEST data for TmDOTA-(gly)_4_^−^ are shown in Fig. 3. To allow for easy comparison of the CEST response of all four LnDOTA-(gly)_4_^−^ complexes, these CEST data are displayed in graphical form in Fig. 4. The CEST effect is markedly pH-dependent showing an increase in CEST with pH values above 6.2 and reaching a maximum around pH 8–8.5. Since the amide proton exchange is accelerated by base, a lack of CEST below pH ∼6.2 indicates that proton exchange is too slow to meet the CEST requirement. As the pH is increased between 6 and 8, the CEST signal gradually increases in intensity for all agents as –NH proton exchange becomes faster. Around pH 8 to 8.5, the rate of proton exchange reaches an optimal value for CEST and above this pH region, -NH proton exchange becomes too fast, and the CEST signal intensity once again decreases as the –NH proton resonance begins to coalesce into the bulk water signal. The pH profile of YbDOTA-(gly)_4_^−^ reported by Aime and co-workers showed a linear pH dependence in the pH range of 5.5–8.1, in reasonable agreement with data presented here for YbDOTA-(gly)_4_^−^ [11].

Among the four lanthanide complexes examined here, the –NH resonance of TmDOTA-(gly)_4_^−^ displayed a 3-fold larger chemical shift than YbDOTA-(gly)_4_^−^ while maintaining a relatively strong CEST signal compared to those of DyDOTA-(gly)_4_^−^ and TbDOTA-(gly)_4_^−^. For these reasons, we chose the Tm(III) complex for encapsulation into liposomes for further CEST amplification.

**Figure 5:**
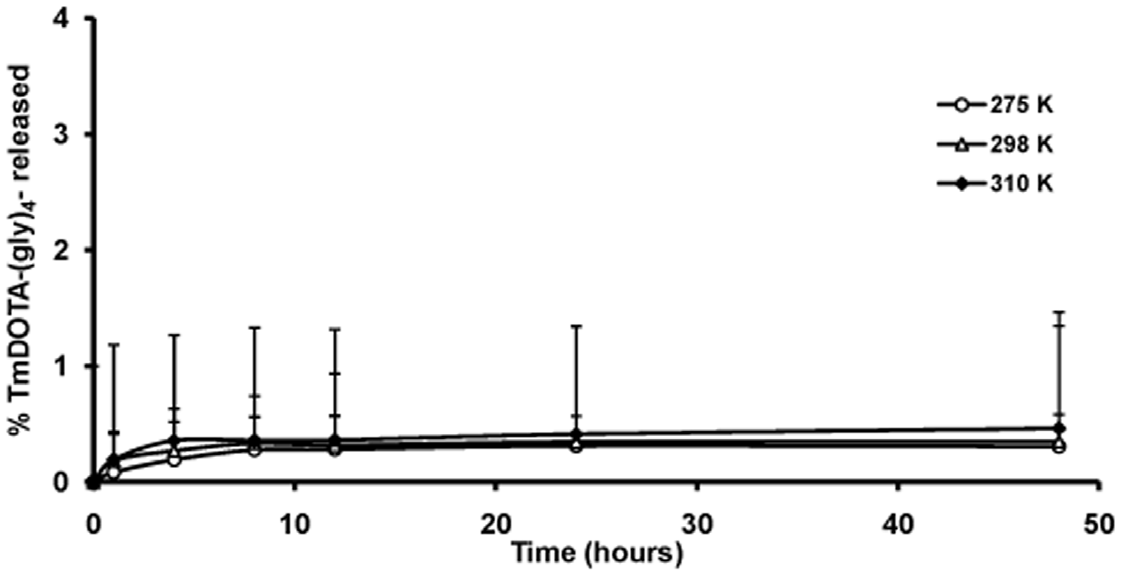
Plot of amount of TmDOTA-(gly)_4_^−^ released from liposomes (%) as a function of time. TmDOTA-(gly)_4_^−^ was measured analytically in the extraliposomal medium during incubation of liposomes at 275 K, 298 K and 310 K over a span of 48 hr.

It is important to show that the liposomes are stable toward leakage of the encapsulated agent at different temperatures and pH values to ensure that the CEST signal reflects agent inside the liposome core and does not arise from agent that may have leaked out. Release of TmDOTA-(gly)_4_^−^ at different temperatures was first evaluated by dialyzing a suspension of liposomes containing the agent against 300 milliosmolar (mOsm) NaCl at three different temperatures, 275 K, 298 K and 310 K. It is important to maintain the same osmolality on both sides of the liposomal bilayer to prevent shrinkage or bursting of the liposomes due to differences in osmotic pressure. Aliquots of dialysate were removed at different time points and analyzed for total Tm(III) using ICP-MS. As shown in Fig. 5, less than 1% of the total TmDOTA-(gly)_4_^−^ loaded into liposomes was released over the 2 day period at the three temperatures examined. These profiles show that the liposome formulation is stable at the temperatures tested.

**Figure 6:**
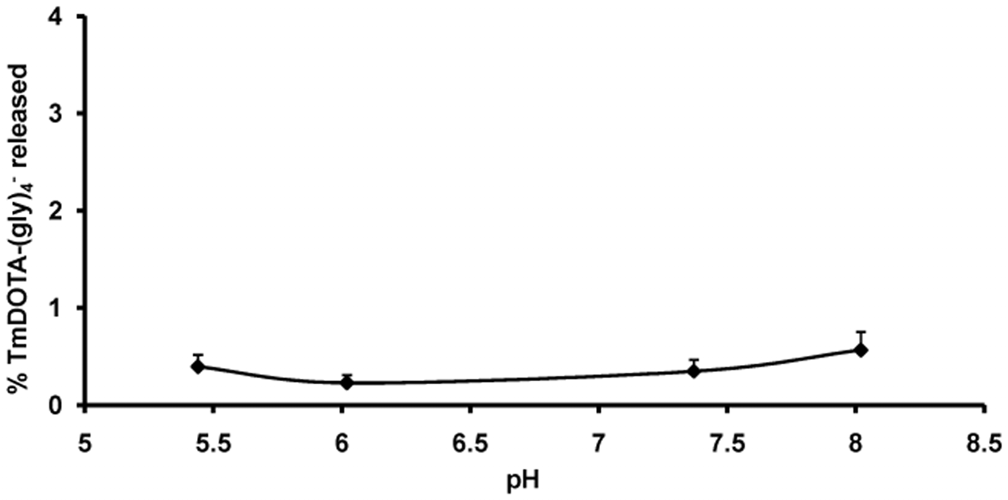
*In vitro* stability of a liposomal suspension at different pH values (n = 2). TmDOTA-(gly)_4_^−^ was measured analytically in the extraliposomal medium after incubation of liposomes at 298 K for 24 hr at several different pH values.

To evaluate the pH stability of the liposomal formulation, the liposomes were suspended at different pH values for 24 hr, then dialyzed against 300 mOsm NaCl at 298 K. Again, any released Tm(III) was monitored using ICP-MS. Figure 6 shows that less than 1% of the total Tm(III) complex leaked from the liposome interior when exposed in acid (pH 5.5) or base (pH 8.0) within 24 hr. Again, this provides evidence for a stable liposome formulation at different pH values.

Prior to pH dependent CEST studies, the amount of entrapped TmDOTA-(gly)_4_^−^ per nanoparticle was optimized by systematically varying the concentration of TmDOTA-(gly)_4_^−^ in the stock solution used during preparation of liposomes from 50–150 mM. It was assumed that the concentration of TmDOTA-(gly)_4_^−^ in the stock solution used in the hydration process was equivalent to the concentration agent entrapped inside the liposome core volume after liposomal preparation and purification. The resulting liposomes had a hydrodynamic diameter of 98–104 nm with low polydispersity index confirming their narrow size distribution (Table 1). Elemental analysis confirmed that liposomes prepared from stock solutions containing 50 mM TmDOTA-(gly)_4_^−^ had the lowest thulium to phospholipid (Tm∶P) ratio while those prepared from solutions containing 150 mM TmDOTA-(gly)_4_^−^ had the highest Tm∶P ratio, as expected.

**Table 1:** Hydrodynamic diameter, polydispersity index and thulium-phosphorus ratio of the liposomes at different concentrations of the encapsulated TmDOTA-(gly)_4_^−^.

[Stock solution of Tm DOTA-(gly)_4_^−^] (mM)	Diameter (nm)	Polydispersity index	Tm∶P
50	101	0.037	0.09
60	104	0.053	0.10
75	98	0.083	0.11
150	103	0.118	0.33

**Figure 7:**
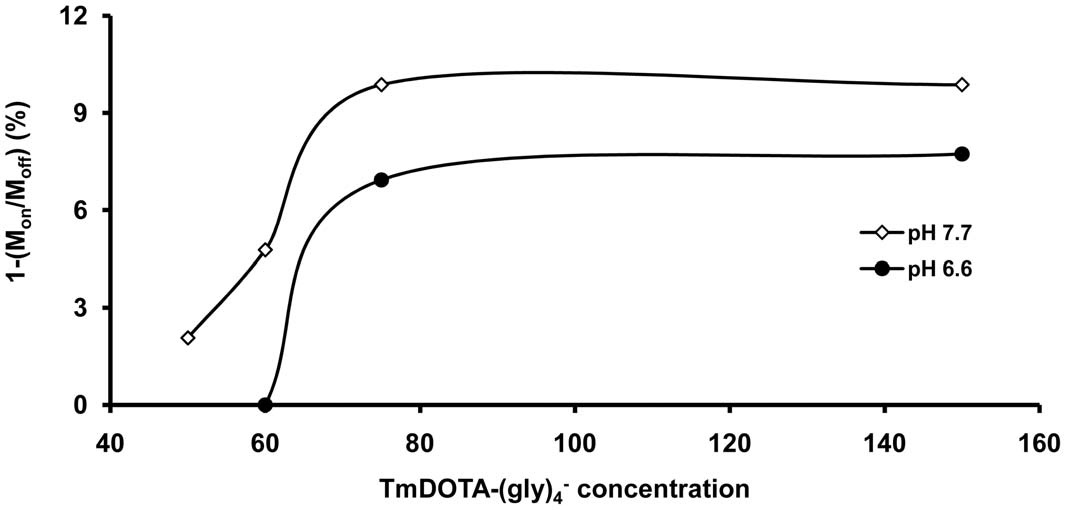
Amide proton CEST intensity from liposomal-encapsulated TmDOTA-(gly)_4_^−^. Each amide proton CEST intensity was measured at the frequency of the exchanging –NH protons (-51 ppm) in an aqueous suspension of liposomes filled with TmDOTA-(gly)_4_^−^ in the inner core (the indicated concentrations were for the stock solutions of TmDOTA-(gly)_4_^−^ used in preparing the liposomes) at 9.4 T and 310 K. (B_1_ = 7.0 µT; presaturation time = 6 s).

Amide proton CEST from liposome-encapsulated TmDOTA-(gly)_4_^−^ was assessed at mild basic (pH 7.7) and mild acidic (pH 6.6) conditions by adjusting the pH using 300 mOsm HEPES or MES buffer. Figure 7 shows a plot of the intensity of amide proton CEST intensity as a function of agent concentration at two different pH values. In all cases, the total bulk solution concentration of TmDOTA-(gly)_4_^−^ in each sample was adjusted to 3 mM by varying the liposome concentration so that liposomes with 150 mM encapsulated agent were 3-fold more dilute (on a liposome basis) compared to samples containing liposomes with 50 mM encapsulated agent. This allowed a direct comparison of each liposome preparation while maintaining an identical bulk concentration of TmDOTA-(gly)_4_^−^ in each sample (3 mM).

As expected for a system undergoing base-catalyzed amide proton exchange, a higher CEST signal was observed at more basic pH values, reaching a plateau of about 10% CEST for encapsulated agent concentrations above ∼75 mM. A similar trend was observed at pH 6.6 but, in this case, the maximum CEST was ∼7%. At agent concentrations below 75 mM, the CEST signal decreased and was not detectable for agent concentrations below ∼40 mM.

**Figure 8:**
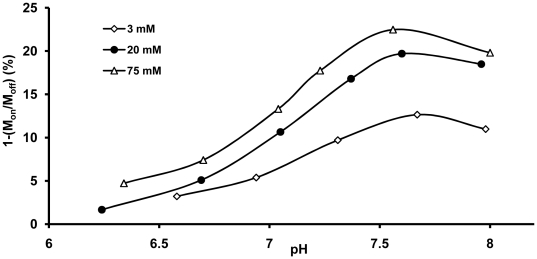
Amide proton CEST intensity *versus* pH for three different concentrations of TmDOTA-(gly)_4_^−^. No lipid was present in this experiment. The amide proton CEST intensity was measured at 9.4 T and 310 K using a B_1_ = 7.0 µT and a presaturation time of 6 s.

Figure 8 compares the amide proton CEST intensity as a function of pH for unencapsulated TmDOTA-(gly)_4_^−^ (no lipid) at three different agent concentrations. The general shape of these pH curves were similar at all three agent concentrations, consistent with base-catalyzed –NH proton exchange. A maximum CEST signal was reached at similar pH values (∼7.6) and, as anticipated, the CEST signal was larger for the sample containing the most agent. However, for an increase in agent concentration from 3 to 20 mM (6.67-fold), the maximum CEST signal at pH 7.6 (the maximum in these curves) only increased from 12 to 20%, a 1.67-fold increase. This indicates that CEST does not vary linearly with agent concentration at pH 7.6. A further increase in agent concentration from 20 to 75 mM resulted only in an additional 2% increase in CEST.

**Figure 9:**
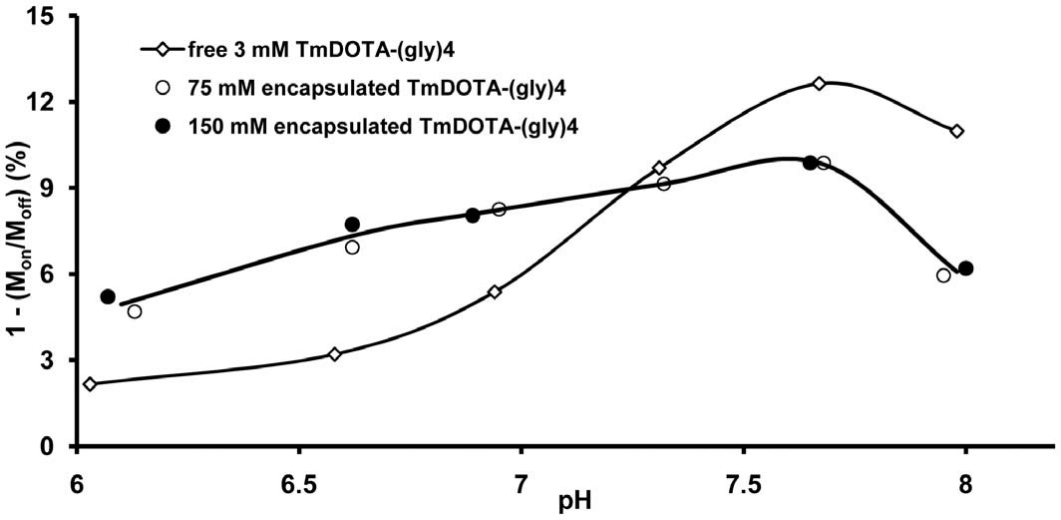
Comparison of amide proton CEST intensities *versus* pH for free and liposome-encapsulated TmDOTA-(gly)_4_^−^. The amide proton CEST intensity for free TmDOTA-(gly)_4_^−^ sample (3 mM, no lipid, same data as that shown in Fig. 8) and liposomal-encapsulated TmDOTA-(gly)_4_^−^ (intraliposomal concentrations of 75 mM and 150 mM) were measured as a function of pH at 9.4 T and 310 K using a B_1_ = 7.0 µT and a presaturation time of 6 s. The number of liposomes in the later two samples was adjusted so that the total concentration of TmDOTA-(gly)_4_^−^ in each sample was 3 mM when averaged over the entire volume.

The pH dependence of amide proton CEST for two different liposome preparations (75 mM and 150 mM encapsulated agents) each at a total TmDOTA-(gly)_4_^−^ concentration of 3 mM are compared with a similar curve for 3 mM unencapsulated TmDOTA-(gly)_4_^−^ (no lipid, same data as shown in Fig. 8) in Figure 9. The CEST versus pH profiles for two encapsulated samples is clearly quite different from that collected for the unencapsulated agent free in solution. Both the free and encapsulated samples reached a maximum CEST intensity near pH ∼7.6 but the CEST profile was considerably flatter and identical for the two encapsulated agents in the lower pH regions. At basic pH values (pH>7), a smaller CEST intensity was observed for the encapsulated TmDOTA-(gly)_4_^−^ samples, likely reflecting the extra barrier for the exchanging protons to equilibrate into the bulk solvent. At lower pH values, the CEST signal from the unencapsulated agent decreases with typical sigmoid behavior to a value approaching zero by pH 6.0. The two encapsulated samples, however, showed a much more modest decrease in CEST intensity at the lower pH values. Interestingly, the encapsulated samples displayed a larger CEST intensity at all pH values below pH∼7.3 as compared to the unencapsulated sample at identical pH values.

## Discussion

The pH sensitivity of the exchangeable amide protons of LnDOTA-tetraamide complexes makes such complexes attractive for pH sensing using CEST principles. In this study, pH dependent CEST curves were presented for four different LnDOTA-(gly)_4_^−^ complexes (where Ln = Yb, Tm, Tb or Dy). The differences in chemical shifts of the amide protons in these complexes were due to the intrinsic magnetic anisotropy of the lanthanide ions, arising from pseudocontact or hyperfine interactions [12]. In addition, the inherently short spin-spin relaxation time (T_2_) of Tm(III) is evidenced by the broadening of the bulk water peak in the CEST spectrum of TmDOTA-(gly)_4_^−^ (Fig. 2).

It is well known that amide proton exchange is accelerated by a base so a change in solution pH results in a change in CEST response. Thus, pH dependent CEST curves were collected for the four different LnDOTA-(gly)_4_^−^ complexes (Fig. 4). Among the four complexes examined, YbDOTA-(gly)_4_^−^ showed the largest CEST intensity at all pH values, followed by TmDOTA-(gly)_4_^−^. The Tb(III) and Dy(III) complexes showed similar and smaller CEST intensities at all pH values. The variation in CEST signal amplitude for the four complexes has been attributed to differences in the intrinsic paramagnetic properties of the lanthanide metal, expressed as the effective magnetic moment (μ_eff_) [9]. A large magnetic moment effectively reduces both T_1_ and T_2_ of water protons and, since the amide proton CEST intensity is in competition with T_1_, a longer T_1_ will allow transfer of more saturated spins before those spins return to equilibrium. Solutions of the Yb(III) complex, being the least paramagnetic (by virtue of its low μ_eff_) among the four lanthanide ions examined here, had the longest T_1_ and this in turn translated to the largest CEST signal (Table 2).

**Table 2:** The effective magnetic moment (μ_eff_) [9] of the four lanthanide ion complexes and the measured T_1_ (sec) ± s.d. of bulk water in 20 mM samples of LnDOTA-(gly)_4_^−^.

Ln	μ_eff_	Bulk water T_1_ (sec) of Ln-1	CEST (%)1-(M_on_/M_off_)
Yb	4.5	1.86±0.01	49
Tm	7.6	0.53±0.01	25
Dy	10.6	0.24±0.01	15
Tb	9.7	0.25±0.01	17

All data were collected at 9.4 T, 310 K on samples adjusted to pH 7.5.

It is important to point out that the decrease in CEST above pH 8–8.5 does not reflect complete dissociation of the amide proton over the pH range studied here but rather changes in amide proton exchange rates over this range of pH values. The pK_a_'s of the amide protons in complexes such as LnDOTA-(gly)_4_^−^ are much higher and typically too high to be easily discerned by pH potentiometry [13], [14]. The present data illustrates that the optimal –NH proton exchange rate for CEST occurs well below the pH at which complete dissociation of an amide proton occurs.

The relatively small chemical shift of the -NH protons in the Yb(III) complex relative to bulk water may pose additional problems *in vivo* because of the added complexities of the background tissue MT signal [15]. One way to minimize interference from the MT signal is to use a LnDOTA-(gly)_4_^−^ complex that has a more highly shifted –NH proton exchange resonance. On the basis of chemical shift and CEST magnitudes of the four complexes studied here, the Tm(III) complex was chosen for encapsulation into liposomes for further CEST studies.

A major limitation of applying CEST agents such as these is their inherently low sensitivity, typically requiring millimolar agent concentrations for detection. One way to improve CEST sensitivity is to amplify the signal by using a nanoparticle assembly such as a liposome. Here, the local concentration of the agent can be amplified substantially by sequestering a large number of lanthanide complexes within the inner core of the nanoparticle. Liposomes can also serve as delivery vehicles for CEST agents to carry a high payload of contrast agent to a site of interest. It has been reported that the inherent leaky vasculature of tumors coupled with the increased capillary permeability of stealth liposomes results in localization of liposomes in tumors without a specific targeting component [16]. This could, in principle, increase the effective concentration of CEST agent at targeted site of interest several fold.

These results stimulated further studies on encapsulation of one or more of these pH reporters in the internal cavity of liposomes. Since the liposomal membrane is semi-permeable to water, the pH of the extra-liposomal environment should be correctly reported by a pH reporter molecule trapped in the interior compartment. The fraction of the internal water in close proximity to the trapped Ln(III) complex is small in comparison to the amount of extra-liposomal water so the interior water molecules and/or protons must exchange with extra-liposomal water to amplify the CEST signal. A liposomal formulation was chosen that consisted of a saturated phospholipid, 1,2-dipalmitoyl-sn-glycero-3-phosphocholine (DPPC), cholesterol and 1,2-distearoyl-sn-glycero-3-phosphoethanolamine-N-[methoxy (polyethylene glycol)-2000] (DSPE-mPEG-2000) in a molar ratio of 55∶40∶5 [17], [18], [19]. Inclusion of saturated phospholipids such as DPPC makes the lipid packing more compact and insertion of cholesterol provides additional stability to the bilayer membrane [20], [21], [22]. Inclusion of lipid derivatives of polyethylene glycol (PEG) in the bilayer was done to prolong the blood circulation time of liposomes by reducing their uptake by mononuclear phagocytes (MPS) [23]. This formulation has been used to encapsulate gadolinium and other multimodal contrast agents where a sustained contrast enhancement due to increased circulation half lives was exhibited by the liposomes in the vasculature of a rat model as compared to the free agents [17], [18]. The size of the liposome was maintained at less than 200 nm to provide longer circulation times and to facilitate proton transport across the bilayer due to higher surface area to volume ratio [17], [24]. Different amounts of TmDOTA-(gly)_4_^−^ were encapsulated in liposomes to determine the optimal agent concentration. The data in Fig. 7 illustrates that maximum CEST is reached with only 75 mM encapsulated TmDOTA-(gly)_4_^−^.

The CEST signal intensity may be described by equation 1 [25], [26].
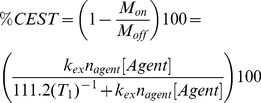
(1)Here, M_on_ and M_off_ are the signal intensities of the bulk water protons at the on- and off-resonance positions, 111.2 is the molar concentration of water protons in solution, and the term 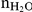
[Agent] refers to the number (n) of exchangeable protons per agent molecule – four in this case. This equation indicates that faster exchange rates (within the limits of the CEST limit k_ex_<Δω), longer water proton spin-lattice relaxation times (T_1_), higher agent concentrations [Agent], and a larger number of exchangeable protons on the agent (n_agent_) all result in a higher CEST signal. This equation also illustrates that CEST is proportional to agent concentration only for those conditions when 111.2(T_1_)^−1^≫k_ex_n_agent_[agent]. At higher agent concentrations, the second term in the denominator dominates and the CEST signal gradually becomes independent of agent concentration, becoming more dependent on other factors such a reduction in water proton T_1_ brought about by the higher concentrations of weakly paramagnetic agent.

Encapsulation of TmDOTA-(gly)_4_^−^ into phospholipid-based liposomes was found to buffer the amide proton CEST intensity *versus* pH response in the more acidic regions as presented in Figure 9. This interesting observation likely reflects a change in membrane permeability of the lipid bilayer at the different pH values. Although zwitterionic phosphocholine (PC) is regarded as an “inert molecule”, studies have shown that acidification of the PC bilayer modifies lipid mobility and consequently the membrane permeability [27]. It has been reported that an increased lipid order and an immobilized acyl chains were observed upon lowering the pH from 7 to 4. At the liquid-crystalline phase or at temperatures near the transition temperature (T_c_) of the phospholipid (Tc of DPPC is 42°C), partial protonation of the PC headgroups may reduce the lipid mobility either by changing the conformational and/or packing changes in the PC headgroup. This change in the lipid bilayer reportedly inhibits release of K^+^ (mediated by an ionophore, valinomycin, which catalyzes release of K^+^), consistent with a decrease in conductance of proton and hydroxide in the PC bilayers [27]. In these preparations, the pH of the original liposomal suspension before addition of buffer was around 7.5. As the pH of the extra-liposomal solution was reduced by addition of acid, the CEST signal remained relatively unchanged (∼3% decrease between pH 7 and 6) but was larger in magnitude at all pH values below 7 than the CEST signal generated by the free, unencapsulated agent. This suggests impedance in the transport of H^+^ across the bilayer as the pH is lowered causing the internal pH to remain closer to the original pH of 7.5 while the pH of the exterior, as monitored by a pH electrode, is lower. One possible way to overcome the proton transport limitation is to render the liposomes less rigid to allow easier transfer of protons across the bilayer. This can be achieved by lowering the cholesterol content in the phospholipid bilayer or by using a less saturated phospholipid. However, one must insure that liposome stability not be compromised by changing the bilayer formulation.

One can compare the sensitivity of a liposome-encapsulated *versus* unencapsulated CEST agent by comparing the concentrations required to produce a 5% decrease in bulk water signal intensity [9], [28]. For this system, 1 mM TmDOTA-(gly)_4_^−^ (no lipid) was required to produce a 5% decrease in water signal while the concentration of liposomes required to produce the same amide proton CEST intensity was ∼100 nM liposome (determined for a preparation that contained 75 mM encapsulated agent). This 10^4^ gain in sensitivity makes responsive lipoCEST agents such as those illustrated here an attractive platform for molecular imaging. Nano-carriers such as these could also be valuable for measuring pH without knowing the exact agent concentration by ratiometric imaging [29]. One could envision entrappment of two different CEST agents (with different exchange frequencies and pH sensitivities) in the interior of a liposome and using the ratio of two CEST spectra to obtain a direct readout of pH. This would also eliminate any concerns about potential differences in tissue biodistribution for the two agents.

## Materials and Methods

The preparation of LnDOTA-(gly)_4_^−^ was previously reported by Aime et al. using an ethyl ester protecting group (8). Here, we report an alternative synthesis using the *tert*-butyl ester protecting group with comparable yields.

### General procedures

All commercially available reagents were used as received, unless stated otherwise. Solution pH was measured using a double junction combination pH electrode (183×3.5 mm, Sigma Aldrich) without temperature correction. ^1^H NMR, ^13^C NMR and CEST spectra were recorded on a Bruker Avance spectrometer (^1^H at 400.13 MHz, ^13^C at 100.61 MHz). Infrared spectra were recorded using a Perkin Elmer 1600 Fourier Transform IR spectrometer. Melting points were recorded on a Fisher/Johns melting point apparatus and are uncorrected.

### Synthesis

#### tert-Butyl-2-(2-bromoacetamido)acetate (2)

Glycine-*tert*-butyl ester hydrochloride (25 g, 148 mmol) and potassium carbonate (103 g, 746 mmol) were dissolved in water (300 mL) and dichloromethane (300 mL) was then added. The biphasic reaction mixture was cooled to 0°C and a solution of bromoacetylbromide (14 mL, 161 mmol) in dichloromethane (140 mL) was added dropwise. The reaction was stirred at 0°C for 30 min, then allowed to warm to room temperature and stirred at this temperature an additional 18 hr. The organic layer was collected and washed with 5% aqueous citric acid (4×100 mL) and water (3×150 mL) before drying over sodium sulfate. The solvent dichloromethane was removed under reduced pressure and the remaining residue was recrystallized in ethyl acetate. The crystals were filtered to afford *tert*-butyl-2-(2-bromoacetamido)acetate (**2**) as a colorless solid (31.0 g, 83%). Mp = 87–88°C. ^1^H-NMR (400 MHz, CDCl_3_): δ 6.97 (1H, s br, NH), 3.97 (2H, d, ^2^J_H-H_ = 5 Hz, NHCH_2_), 3.91 (2H, s, CH_2_Br), 1.49 (9H, s, C(CH_3_)_3_). ^13^C-NMR (400 MHz, CDCl_3_): δ 168.3 (CO_2_), 165.7 (NHC = O), 82.7 (C(CH_3_)_3_), 42.5 (NHCH_2_CO), 28.6 (BrCH_2_), 28.00 (C(CH_3_)_3_). IR ν_max_/cm^−1^ (KBr disk): 3261 (NH), 3087, 2978, 1738 (C = O), 1651 (C = O), 1574, 1410, 1378, 1228, 1176, 1039, 664. Anal. calcd for C_8_H_14_BrNO_3_: C, 38.1; H, 5.6; N 5.6. Found: C, 36.7, H, 5.1; N, 5.9.

#### 1,4,7,10- tetraazacyclododecane-1,4,7,10-tetra-(tert-butyl-acetamidoacetate) (3)

Cyclen (850 mg, 5.00 mmol) and potassium carbonate (3.63 g, 26 mmol) were suspended in acetonitrile (200 mL) and stirred for 20 minutes at 60°C. Bromoacetamide **2** (5.16 g, 20 mmol) was added and the reaction mixture was stirred at 60°C for 3 days and then allowed to cool to room temperature. The mixture was filtered and the solvents were removed *in vacuo* to give an oily residue. The residue was taken up in dichloromethane (250 mL) and washed with brine (3×100 mL). The organic extracts were collected, dried over Na_2_SO_4_, and solvent dichloromethane was removed *in vacuo* to afford 1,4,7,10- tetraazacyclododecane-1,4,7,10-tetra-(tert-butyl-acetamidoacetate) (**3**) as a pale yellow hygroscopic solid (4.0 g, 83%). Mp: 185–186°C. ^1^H-NMR (400 MHz, CDCl_3_): δ 7.48 (4H, s br, NH), 3.83 (8H, d, ^2^J_H-H_ = 6 Hz, HNCH_2_CO), 3.09 (8H, s, NCH_2_CO), 2.68 (16H, s, ring NCH_2_), 1.38 (36H, s, C(CH_3_)_3_). ^13^C-NMR (400 MHz, CDCl_3_): δ 171.3 (CO_2_), 169.1 (CONH), 81.9 (C(CH_3_)_3_), 59.2 (NCH_2_CO), 53.4 (ring NCH_2_), 41.6 (NHCH_2_CO), 28.0 (C(CH_3_)_3_). IR ν_max_/cm^−1^ (KBr disk): 3202 (NH), 2976, 2836, 1747 (C = O), 1670 (C = O), 1551, 1457, 1369, 1225, 1159, 1034. *m/z* (ESMS EI+): 879 (100%, [M+Na^+^], Anal. Calcd for C_40_H_72_N_8_O_12_.KBr: C, 49.2; H, 7.4; N 11.5. Found: C, 50.5, H, 7.3; N, 11.6.

#### 1,4,7,10- tetraazacyclododecane-1,4,7,10-tetraacetamidoacetate (1)

Excess trifluoroacetic acid (TFA) was added to *tert*-butyl ester **2** (3.75 g, 0.00438 mol) and stirred for 18 hours. TFA was then removed *in vacuo* and the oily residue was dissolved in diethyl ether (40 mL) and stirred for 30 min. The white precipitate that formed was filtered and washed with cold diethyl ether (2×20 mL). The solid precipitate was then taken up in water (30 mL) and lyophilized. DOTA-(gly)_4_ (**1**) was obtained as a colorless solid (4.1 g, 83%). Mp: 89–92°C. ^1^H-NMR (400 MHz, D_2_O): δ3.61 (8H, s, NHCH_2_CO), 3.06 (8H, s, NCH_2_CO), 2.55 (16H, s, ring NCH_2_). ^13^C-NMR (400 MHz, D_2_O): δ176.8 (CO_2_), 173.4 (CONH), 57.1 (NCH_2_CO), 50.8 (ring NCH_2_), 43.1 (NHCH_2_CO). IR ν_max_/cm^−1^ (KBr disk): 3346 (OH), 2971, 2723, 1734 (C = O), 1684 (C = O), 1559, 1456, 1379, 1189, 1034. *m/z* (ESMS EI+): 633 (67%, [M+H^+^]), 655 (100%, [M+Na^+^]). Anal. Calcd for C_24_H_40_N_8_O_12_.3CF_3_COOH.H_2_O.KBr: C, 33.0; H, 4.4; N 9.9. Found: C, 33.1, H, 4.1; N, 9.6.

### Preparation of lanthanide complexes

LnDOTA-(gly)_4_^−^ complexes were prepared by mixing an equimolar amount (0.643 mmol) of the ligand and the corresponding lanthanide chloride (Ln = Tb, Dy, Tm, Yb) in 5 mL of water. The pH of the solution was maintained near pH 6 using 3 N NaOH at room temperature. The progress of the lanthanide complexation was followed by reverse-phase HPLC using a Phenomenex Luna amino (NH_2_) column, 5 µm (150×3.0 mm). The absorbance was monitored at 210 nm and the solvent system elution started with 100% acetonitrile∶water (15∶85) followed by a linear gradient to 15% acetonitrile∶water (15∶85) and 85% 20 mM phosphate buffer (pH 7.0) over 15 min and was further maintained for 5 min, at a flow rate of 1 mLmin^−1^ (chelate *t*_R_ = 4.68 min; ligand *t*_R_ = 15.84 min). The complex was concentrated by lyophilization then redissolved in a minimum amount of methanol∶water (30∶10). Tetrahydrofuran was added to the solution dropwise until precipitate began to form and turbidity persisted. The mixture was stirred for 17 hr in an ice bath followed by centrifugation. Lyophilization of the precipitate yielded a white solid.

#### TmDOTA-(gly)_4_^−^

^1^H NMR (400 MHz, in H_2_O at pH 7.50, 25°C, ref. δH_2_O = 0 ppm): δ 253.3 (4H, s, ring NCH_2_, axial), 37.7 (4H, s, ring NCH_2_, equatorial), 27.6 (4H, s, 4H, ring NCH_2_, equatorial), −9.6 (4H, s, NHCH_2_CO), −19.7 (4H, s, NHCH_2_CO), −55.1 (4H, s br, NH), −81.7 (4H, s, NCH_2_CO), −93.3 (4H, s, ring NCH_2_, axial), −169.2 (4H, s, NCH_2_CO). *m/z* (ESMS EI+): 843 (100%, [M^−^+2Na^+^]^+^).

#### YbDOTA-(gly)_4_^−^

^1^H-NMR (400 MHz, in H_2_O at pH 7, 25°C, ref. δH_2_O = 0 ppm): δ 92.4 (4H, s, ring NCH_2_, axial), 13.2 (4H, s, ring NCH_2_, equatorial), 10.2 (4H, s, ring NCH_2_, equatorial), −4.4 (4H, s, NHCH_2_CO), −8.3 (4H, s, NHCH_2_CO), −17.4 (4H, s br, NH), −30.5 (4H, s, NCH_2_CO), −35.9 (4H, s, ring NCH_2_, axial), −62.2 (4H, s, NCH_2_CO). *m/z* (ESMS EI+): 848 (100%, [M^−^+2Na^+^]^+^), 879 (27%, [M^−^+2K^+^]^+^).

#### TbDOTA-(gly)_4_^−^

^1^H-NMR (400 MHz, in H_2_O at pH 7, 25°C, ref. δH_2_O = 0 ppm): δ 203.7 (4H, s, NCH_2_CO), 103.3 (4H, s, ring NCH_2_, axial), 66.7 (4H, s br, NH), 54.1 (4H, s, NCH_2_CO), 29.0 (4H, s, NHCH_2_CO), 22.8 (4H, s, NHCH_2_CO), −86.1 (4H, s, ring NCH_2_, equatorial), −88.5 (4H, s, ring NCH_2_, equatorial), −320.9 (4H, s, ring NCH_2_, axial). *m/z* (ESMS EI+): 833 (100%, [M^−^+2Na^+^]^+^).

#### DyDOTA-(gly)_4_^−^

^1^H-NMR (400 MHz, in H_2_O at pH 7, 25°C, ref. δH_2_O = 0 ppm): δ 240.7(4H, s, NCH_2_CO), 124.3 (4H, s, ring NCH_2_, axial), 82.4 (4H, s br, NH), 76.6 (4H, s, NCH_2_CO), 32.6 (4H, s, NHCH_2_CO), 24.0 (4H, s, NHCH_2_CO), −89.2 (8H, s, ring NCH_2_, equatorial), −375.7 (4H, s, ring NCH_2_, axial). *m/z* (ESMS EI+): 837 (100%, [M^−^+2Na^+^]^+^).

### CEST experiments

NMR samples for CEST studies were prepared by adding 0.10 M LnDOTA-(gly)_4_^−^ (100 µL) in 10 mM 2-(N-morpholino)ethanesulfonate (MES) buffer for pH 5.5–6.5, 4-(2-hydroxyethyl)-1-piperazineethanesulfonate (HEPES) buffer for pH 6.5–8.5 and Tris buffer for pH 8.5 to 9.5 (400 µL). LipoCEST samples were prepared by adding 1.41 µM liposome encapsulating monosodium LnDOTA-(gly)_4_ (300 µL) in 10 mM 2-(N-morpholino)ethanesulfonate (MES) buffer for pH 5.5–6.5, 4-(2-hydroxyethyl)-1-piperazineethanesulfonate (HEPES) buffer for pH 6.5–8.5 and Tris buffer for pH 8.5–9.5 plus an additional amount of NaCl to bring the total solution osmolarity to 300 mOsm (200 µL). The experiment was carried out by irradiating the sample with a continuous wave presaturation pulse at a power level of 21.2, 16.5, 11.8 or 7.0 µT for 2–4 s (2 s for TmDOTA-(gly)_4_^−^, TbDOTA-(gly)_4_^−^, DyDOTA-(gly)_4_^−^ and 4 s pulse duration for YbDOTA-(gly)_4_^−^) over a range of frequencies followed by a single observe pulse to measure the residual water signal. The CEST signal was defined by difference in intensities of the bulk water signal after a presaturation pulse set to the frequency of the exchanging –NH proton (M_on_) minus at the same offset frequency but on the opposite side with respect to the bulk water signal (M_off_). This method removes any effects due to indirect saturation of the bulk water signal.

### Preparation of liposomes

A lipid mixture of 1,2-dipalmitoyl-sn-glycero-3-phosphocholine (DPPC) (Avanti Polar Lipids, Inc., Alabama, USA), Cholesterol (Avanti PolarLipids, Inc., Alabama, USA) and 1,2-distearoyl-sn-glycero-3-phosphoethanolamine-N-[methoxy (poly(ethylene glycol))-2000] (DSPE-mPEG2000) (Avanti PolarLipids, Inc., Alabama, USA) in the molar ratio of 55∶40∶5 was dissolved in absolute ethanol (volume of absolute ethanol was 10% of the total batch volume) at 50°C. A solution of the lanthanide complex (40 mM) was subsequently added to the ethanol solution to achieve a lipid concentration of 150 mM. The solution was stirred for 90 minutes at 50°C and then sequentially extruded using a Lipex Thermoline extruder (Northern Lipids, Vancouver, British Columbia, Canada) with five passes through a 0.20-µm polycarbonate membrane filter and 10 passes through a 0.10-µm polycarbonate membrane filter. To remove the unencapsulated lanthanide complex, the liposomal suspension was diafiltered against 300 mOsm NaCl solution using a MicroKros module (Spectrum Laboratories, California, USA) with a 500 kDa molecular weight cut-off.

### Particle size and composition of liposomal agents

The size distribution of the resulting liposomes was determined by dynamic light scattering (DLS) (Brookhaven 90 Plus DLS Particle Size Analyzer) at 25°C. The lanthanide and phosphorus concentration was quantified using inductively coupled plasma mass spectrometer (ICP-MS, Perkin-Elmer SCIEX ELAN 6100 DRC).

### Liposome stability at different temperatures

The liposomal formulation (100 µL) was re-suspended in 300 mOsm NaCl (1 mL) and was placed in a dialysis bag (10,000 molecular weight cut-off) and dialyzed against 300 mOsm NaCl (300 mL) at different temperatures (4°C, 25°C and 37°C). At given time points, 1 mL of the dialysate was extracted and then replaced with fresh buffer. The released lanthanide complex was quantified using ICP-MS on the withdrawn aliquots.

### Liposome stability at different pH values

Liposomes containing the encapsulated LnDOTA-(gly)_4_^−^ (100 µL) were added to 1 mL of 300mOsm 2-(N-morpholino)ethanesulfonate (MES) buffer for pH pH 5.5–6.5, 4-(2-hydroxyethyl)-1-piperazineethanesulfonate (HEPES) buffer for pH 6.5–8.5 or TRIS buffer for pH 8.5 to 9.5. The resulting liposomal suspensions were incubated at 37°C for 24 hours. The amount of released lanthanide complex at different pH was determined by dialyzing each of the samples against 300 mOsm NaCl (300 mL) at 25°C and the dialysate was analyzed using ICP-MS.

## References

[1] Lauffer RB (1987). Paramagnetic Metal-Complexes as Water Proton Relaxation Agents for Nmr Imaging - Theory and Design.. Chemical Reviews.

[2] Wolff SD, Balaban RS (1989). Magnetization Transfer Contrast (Mtc) and Tissue Water Proton Relaxation Invivo.. Magnetic Resonance in Medicine.

[3] Wolff SD, Balaban RS (1990). Nmr Imaging of Labile Proton-Exchange.. Journal of Magnetic Resonance.

[4] Wolff SD, Eng J, Balaban RS (1991). Magnetization Transfer Contrast - Method for Improving Contrast in Gradient-Recalled-Echo Images.. Radiology.

[5] Aime S, Barge A, Botta M, De Sousa AS, Parker D (1998). Direct MMR spectroscopic observation of a lanthanide-coordinated water molecule whose exchange rate is dependent on the conformation of the complexes.. Angewandte Chemie-International Edition.

[6] Zhang SR, Winter P, Wu KC, Sherry AD (2001). A novel europium(III)-based MRI contrast agent.. Journal of the American Chemical Society.

[7] Aime S, Castelli DD, Terreno E (2005). Highly sensitive MRI chemical exchange saturation transfer agents using liposomes.. Angewandte Chemie-International Edition.

[8] Aime S, Barge A, Castelli DD, Fedeli F, Mortillaro A (2002). Paramagnetic lanthanide(III) complexes as pH-sensitive chemical exchange saturation transfer (CEST) contrast agents for MRI applications.. Magnetic Resonance in Medicine.

[9] Terreno E, Castelli DD, Cravotto G, Milone L, Aime S (2004). Ln(III)-DOTAMGIY complexes: A versatile series to assess the determinants of the efficacy of paramagnetic chemical exchange saturation transfer agents for magnetic resonance imaging applications.. Investigative Radiology.

[10] Sherry AD, Caravan P, Lenkinski RE (2009). Primer on Gadolinium Chemistry.. Journal of Magnetic Resonance Imaging.

[11] Aime S, Barge A, Castelli DD, Fedeli F, Mortillaro A (2002). Paramagnetic lanthanide(III) complexes as pH-sensitive chemical exchange saturation transfer (CEST) contrast agents for MRI applications.. Magn Reson Med.

[12] Bleaney B (1972). Nuclear Magnetic-Resonance Shifts in Solution Due to Lanthanide Ions.. Journal of Magnetic Resonance.

[13] Baranyai Z, Brucher E, Ivanyi T, Kiraly R, Lazar I (2005). Complexation properties of N,N′,N″,N′″-[1,4,7,10-tetraazacyclododecane-1,4,7,10-tetrayltetrakis(1-oxoethane-2,1-diyl)]tetrakis[glycine] (H(4)dotagl). Equilibrium, kinetic, and relaxation behavior of the lanthanide(III) complexes.. Helvetica Chimica Acta.

[14] Baranyai Z, Banyai I, Brucher E, Kiraly R, Terreno E (2007). Kinetics of the formation of [Ln(DOTAM)](3+) complexes.. European Journal of Inorganic Chemistry.

[15] Eng J, Ceckler TL, Balaban RS (1991). Quantitative H-1 Magnetization Transfer Imaging Invivo.. Magnetic Resonance in Medicine.

[16] Allen TM (1994). Long-Circulating (Sterically Stabilized) Liposomes for Targeted Drug-Delivery.. Trends in Pharmacological Sciences.

[17] Ayyagari AL, Zhang XD, Ghaghada KB, Annapragada A, Hu XP (2006). Long-circulating liposomal contrast agents for magnetic resonance imaging.. Magnetic Resonance in Medicine.

[18] Zheng JZ, Liu JB, Dunne M, Jaffray DA, Allen C (2007). In vivo performance of a liposomal vascular contrast agent for CT and MR-based image guidance applications.. Pharmaceutical Research.

[19] Ghaghada KB, Bockhorst KHJ, Mukundan S, Annapragada AV, Narayana PA (2007). High-resolution vascular imaging of the rat spine using liposomal blood pool MR agent.. American Journal of Neuroradiology.

[20] Kirby C, Clarke J, Gregoriadis G (1980). Effect of the Cholesterol Content of Small Unilamellar Liposomes on Their Stability Invivo and Invitro.. Biochemical Journal.

[21] Gregoriadis G, Davis C (1979). Stability of Liposomes Invivo and Invitro Is Promoted by Their Cholesterol Content and the Presence of Blood-Cells.. Biochemical and Biophysical Research Communications.

[22] De Gier J, Mandersloot JG, Van Deenen LLM (1968). Lipid composition and permeability of liposomes.. Biochimica Et Biophysica Acta.

[23] Klibanov AL, Maruyama K, Torchilin VP, Huang L (1990). Amphipathic Polyethyleneglycols Effectively Prolong the Circulation Time of Liposomes.. Febs Letters.

[24] Zhao JM, Har-El YE, McMahon MT, Zhou J, Sherry AD (2008). Size-induced enhancement of chemical exchange saturation transfer (CEST) contrast in liposomes.. Journal of the American Chemical Society.

[25] Woessner DE (1961). Nuclear Transfer Effects in Nuclear Magnetic Resonance Pulse Experiments.. Journal of Chemical Physics.

[26] Mcconnell HM (1958). Reaction Rates by Nuclear Magnetic Resonance.. Journal of Chemical Physics.

[27] Massari S, Folena E, Ambrosin V, Schiavo G, Colonna R (1991). Ph-Dependent Lipid Packing, Membrane-Permeability and Fusion in Phosphatidylcholine Vesicles.. Biochimica Et Biophysica Acta.

[28] Aime S, Delli Castelli D, Terreno E (2002). Novel pH-reporter MRI contrast agents.. Angewandte Chemie-International Edition.

[29] Ward KM, Balaban RS (2000). Determination of pH using water protons and chemical exchange dependent saturation transfer (CEST).. Magnetic Resonance in Medicine.

